# Matrix Hyaluronan-CD44 Interaction Activates MicroRNA and LncRNA Signaling Associated With Chemoresistance, Invasion, and Tumor Progression

**DOI:** 10.3389/fonc.2019.00492

**Published:** 2019-06-21

**Authors:** Lilly Y. W. Bourguignon

**Affiliations:** Endocrine Unit (111N2), Department of Medicine, San Francisco Veterans Affairs Medical Center, University of California, San Francisco, San Francisco, CA, United States

**Keywords:** hyaluronan (HA), CD44, miRNAs, LncRNA UCA1, chemoresistance, invasion, tumor progression

## Abstract

Tumor malignancies involve cancer cell growth, issue invasion, metastasis and often drug resistance. A great deal of effort has been placed on searching for unique molecule(s) overexpressed in cancer cells that correlate(s) with tumor cell-specific behaviors. Hyaluronan (HA), one of the major ECM (extracellular matrix) components have been identified as a physiological ligand for surface CD44 isoforms which are frequently overexpressed in malignant tumor cells during cancer progression. The binding interaction between HA and CD44 isoforms often stimulates aberrant cellular signaling processes and appears to be responsible for the induction of multiple oncogenic events required for cancer-specific phenotypes and behaviors. In recent years, both microRNAs (miRNAs) (with ~20–25 nucleotides) and long non-coding RNAs (lncRNAs) (with ~200 nucleotides) have been found to be abnormally expressed in cancer cells and actively participate in numerous oncogenic signaling events needed for tumor cell-specific functions. In this review, I plan to place a special emphasis on HA/CD44-induced signaling pathways and the presence of several novel miRNAs (e.g., miR-10b/miR-302/miR-21) and lncRNAs (e.g., UCA1) together with their target functions (e.g., tumor cell migration, invasion, and chemoresistance) during cancer development and progression. I believe that important information can be obtained from these studies on HA/CD44-activated miRNAs and lncRNA that may be very valuable for the future development of innovative therapeutic drugs for the treatment of matrix HA/CD44-mediated cancers.

## Introduction

Cancer cells are known to display dysregulated signaling pathways which are responsible for abnormal cellular functions (1–3). Myriad studies have attempted to understand the cellular and molecular mechanisms involved in the onset of tumor cell-specific behaviors (e.g., tumor cell migration, invasion, survival, and chemoresistance). Interactions between matrix hyaluronan (HA), the major glycosaminoglycan component of extracellular matrix (ECM), and variant isoforms of CD44 (HA receptor) have been shown to be tightly linked to the development of aberrant signaling events in a variety of cancers (4, 5, 5–30). It is known that HA binding to certain isoforms of CD44 selectively activates multiple oncogenic signaling pathways leading to tumor cell-specific phenotypes (4, 5, 5–30). HA is also present in different sizes (e.g., large vs. small sizes) (4, 5, 5–28). The binding interaction between large size HA-CD44 and small size HA-CD44 may cause selective activation of downstream effector functions in cancer stem cells (31–35). Furthermore, recent studies indicate that HA-CD44 interaction stimulates the expression of specific microRNAs (miRNAs) and coordinates downstream, intracellular signaling pathways that influence multiple tumor cell-specific functions (31–35). This review focuses first on matrix HA interaction with CD44 in regulating cancer cell signaling pathways, and then describes downstream target functions of these signaling events that contribute to tumor initiation, migration, invasion, chemoresistance, and tumor progression. We believe that this new information could establish the ground work for developing novel therapeutic agents that would effectively target HA/CD44-activated signaling events and specific downstream target molecules/functions in tumor cells-thus providing important new cancer therapies.

## Matrix Hyaluronan (HA) in Cancers

**Figure 1:**
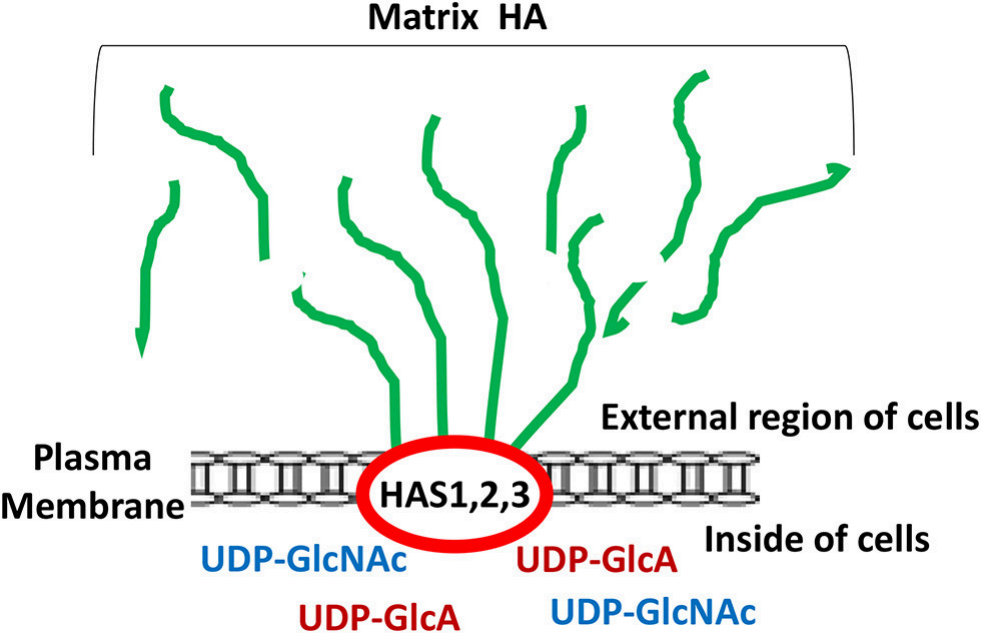
Illustration of matrix hyaluronan (HA) structure regulated by HA synthases (HAS1, HAS2, and HAS3). Green lines represent matrix HA which is made by HAS1, HAS2, and HAS3 followed by being secreted into the external region of the cells.

It has been well accepted fact that unique oncogenesis-induced migration, invasion and metastasis of tumor cells play key roles in causing morbidity in patients (1–3). Many studies have searched for unique molecules which are frequently expressed by cancer cells which correlate with tumor-specific properties. Matrix hyaluronan (HA) known to consist of both D-glucuronic acid and N-acetyl-D-glucosamine in a form of repeating disaccharide units in the extracellular matrix (ECM) (4–7) has been recognized as one of the important contributors in causing tumor development and progression (5, 8–11). It is well-documented that HA is first made by two precursor molecules, uridine diphosphate-glucuronic acid (UDP-GlcA) and uridine diphosphate N-acetylglucosamine (UDP-GlcNAc) through the regulation of HAS1, HAS2, and HAS3 (also known as HA synthase enzymes) inside of the cells and then becomes secreted into the external environment (outside of the cells) as one of the major ECM components in both normal and malignant cells (Figure 1). Generation of large sizes of HA polymers (>2–3 × 10^6^ Daltons) often requires HAS1 and HAS2, whereas the production of smaller-size of HA (<1–2 × 10^5^ Daltons) appears to rely on HAS3 (8–10). A few oncogenic signaling events have been shown to be involved in the unusual activities of HAS1, HAS2, and HAS3 and cause aberrant synthesis and production of HA which then promotes changes of cellular functions and onset of malignant transformation and cancer development (8–10). Large sizes of HA often can be degraded into many biologically active mid-sized and/or small-sized fragments by hyaluronidases such as Hyal-1, Hyal-2, or PH20/Spam1 (11). Most importantly, the level of HA appears to be elevated at the contact region between cancer cells and extracellular matrix (ECM) which may be responsible for the induction of cancer cell-associated properties (5). Thus, over production of HA may be used as a predictor of cancer development.

It has been well documented that HA promotes a variety of oncogenic signaling pathways and causes abnormal physiological changes in cancer cells. For example, HA activates PI3K-AKT signaling pathway which is known to be responsible for tumor cell proliferation, glucose metabolism, cytokine production, angiogenesis and survival (36, 37). Overproduction of HA often induces certain metabolic changes such as accelerating the hexosamine biosynthetic pathway and glycolysis process in breast cancer cells (38). There is also growing evidence that treatment of cancer cells with HA upregulates the expression of the multidrug transporter, *MDR1* (P-glycoprotein), and ABC drug transporters (ABCB3, ABCC1, ABCC2, and ABCC3) leading to aberrant drug fluxes and chemoresistance in breast and ovarian cancer cells (39, 40). Most importantly, HA activates cytoskeleton regulators such as RhoGTPases (e.g., Rho, Rac, and Cdc42) which are known to regulate tumor cell migration, and invasion (41). Additionally, HA is capable of upregulating Rho-kinase activities which in turn stimulates 1,4,5-triphosphate (IP3)-mediated Ca2+ fluxes and endothelial cell migration-a required step for angiogenesis (42, 43). Moreover, certain sizes of low molecular weight hyaluronan appears to induce angiogenesis involving Cdc42 signaling (44). Thus, these findings suggest that abnormal HA-mediated signaling processes may play a critical role in regulating tumor cell-specific properties. To further dissect the cellular and molecular mechanisms involved in HA-mediated oncogenesis, we decided to focus on the interaction between HA and its binding receptor, CD44, in a variety of cancer cells as described below.

## CD44 in Cancers

**Figure 2:**
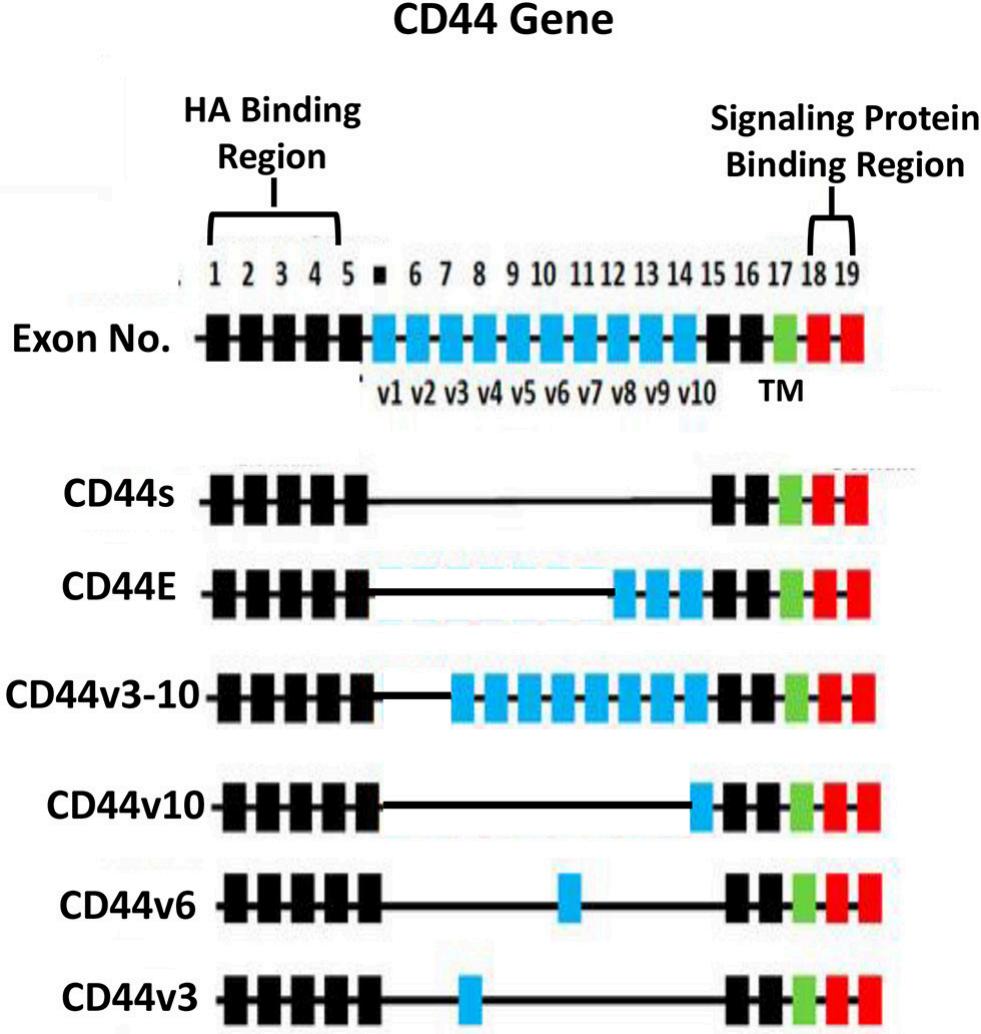
Illustration of CD44 gene, CD44s (the standard form) and alternative spliced variants (CD44E, CD44v3-10, CD44v10, CD44v6, and CD44v3 isoforms). The HA binding domain is located at the external (N-terminal exon 1–5) region of all CD44 isoforms and the signaling protein binding sites are located at the cytoplasmic domain (exon 18–19) of CD44 isoforms. All isoforms contain a transmembrane domain (TM) (exon 17).

HA binding receptor, CD44 is a transmembrane glycoprotein and has been detected in both normal and tumor cells (12–16). Importantly, upregulation of CD44 is often closely associated with abnormal tumor cell behaviors (e.g., proliferation, survival, migration/invasion, and chemoresistance) (13–15). Based on the results from nucleotide sequence analyses, CD44 appears to be encoded by a single gene with 19 exons and exhibits in many different isoforms (16, 17). For example, CD44s (so-called CD44 standard form), contains exons 1–5 at the N-terminal region (with HA binding sites), exons 15–16 at the membrane proximal area and exon 17 at the transmembrane region, as well as exons 18–19 at C-terminal region (with signaling regulation capacity) (Figure 2). CD44 is also known to undergo alternative spicing processes (16, 17). Potentially, the alternative splicing events can occur at 12 exons (out of the 19 exons). Frequently, it has been observed that different exons become inserted at the external region near the membrane proximal domain (between exon 6-14 or v1-v10) of CD44 (16, 17) (Figure 2). For example, exons 12 (v8), 13 (v9), and 14 (v10) are inserted into the CD44s transcripts in epithelial cells (18, 19). Additional exon 7-14 (v3-v10) and exon 14 (v10) have been found to be inserted into the CD44s transcript in keratinocytes and endothelial cells, respectively (20, 21) and these isoforms have been designated as CD44v10 and CD44v3-10 (20, 21) (Figure 2). Most of these CD44 variant (CD44v) isoforms share similar HA binding capacity at the N-terminal region of CD44 (exon 1-5) and a transmembrane domain (exon 17) as well as a signaling interactive region at the cytoplasmic site (exon 18–19). The differences of CD44v isoforms appear to occur at the membrane proximal region (exon 6–14) of the CD44 molecules. A variety of unique CD44 isoforms have been detected in cancer cells and tumor samples (18, 22–28). Thus, selective expression of CD44v isoforms may be considered as a useful bio-marker for the detection of a variety of cancers (18, 22–28).

CD44 isoforms have also been detected in cancer stem cells (CSCs) which appear to display unique ability to initiate tumor cell-specific properties (29–33). For example, tumor cells with high expression of CD44 (but not cells with low CD44 expression) have been shown to induce the formation of tumors in animals with a small numbers of tumor cell injection (29, 30). In head and neck cancer, tumors also contain a cell subpopulation characterized by a high level of CD44v3 expression (29, 30). Furthermore, injection of cells with a high level of CD44v3 expression into immunodeficient mice has been shown to induce multiple types of phenotypically distinct cells, resulting in heterogeneous tumors (31–33). Thus, CD44 isoforms may be used as an important tumor marker for the detection of CSCs. Most importantly, HA-CD44 interaction stimulates CSC downstream signaling processes leading to cancer cell properties and tumor progression (14, 15, 19, 31–33, 35, 36).

A previous study found that CD44 is frequently located in specialized microdomains in the plasma membrane, so-called lipid rafts of cancer cells (45). The binding of HA to CD44 recruits Na+-H+ exchanger (NHE1) and Hyal-2 into CD44-containing lipid rafts, leading to both intracellular and extracellular acidification, HA modification, cathepsin B activation, and breast tumor cell invasion (i). In endothelial cells CD44v10 also interacts with the membrane-associated cytoskeletal protein, ankyrin and an intracellular calcium channel IP3 receptor in the lipid raft (43). These events result in endothelial cell adhesion and proliferation (43). Another study indicated that HA binding to CD44 promotes recruitment of adaptor and/or linker molecules with CD44 in cancer cells. For example, HA induces CD44v3-Vav2 (a guanine nucleotide exchange factor) and Grb2-p185(HER2) complex formation which then causes the co-activation of both Rac1 and Ras signaling leading to the concomitant onset of tumor cell growth and migration required for ovarian tumor progression (46). In addition, HA induces CD44 interaction with a RhoA-specific guanine nucleotide exchange factor (leukemia-associated RhoGEF (LARG) in human head and neck squamous carcinoma cells (47). This event results in Rho/Ras co-activation leading to PLC epsilon-Ca2+ signaling, and Raf/ERK up-regulation required for CaMKII-mediated cytoskeleton function in head and neck squamous cell carcinoma progression (47). Moreover, HA stimulates CD44 interaction with the transforming growth factor beta (TGF-beta) receptors (a family of serine/threonine kinase membrane receptors) in human metastatic breast tumor cells (MDA-MB-231 cell line). This interaction promotes activation of multiple signaling pathways leading to membrane-cytoskeleton interaction, tumor cell migration, and important oncogenic events (e.g., Smad2/Smad3 phosphor and PTH-rP production) during HA and TGF-beta-mediated metastatic breast tumor progression (48). Additionally, it has been observed that HA induces CD44 interaction with RHAMM (receptor of HA-mediated motility) and causes cell motility, increased wound healing, and modification of signal transduction of the Ras signaling cascade (49–51). Furthermore, there is a report showing low molecular weight HA induces CD44 interaction with toll-like receptors. This signaling event then promotes the actin filament-associated protein 110-actin binding and MyD88-NFκB signaling resulting in proinflammatory cytokine/chemokine production and breast tumor invasion (52). Together these findings strongly suggest that the interaction between HA/CD44 and a variety of different membrane proteins and/or regulatory molecules plays a pivotal role in regulating solid tumor cancer progression.

## HA-CD44 Interaction in Promoting MicroRNA Signaling and Tumor Progression

A class of 21–25 nucleotide length small RNAs, so called microRNAs (or miRNAs) have been shown to be involved in gene regulation (53). Overall, the impact of miRNA-regulated gene expression appears to be significant since specific miRNAs may influence the downstream effector gene expression and functions (53). For example, at least four miRNA clusters, such as let-7a-d, let-7i, miR-15b-16-2, and miR-106b-25, have been identified as being involved in G1-S transition (54) during cell cycle progression and tumor progression (55, 56). Moreover, dysregulation of certain miRNAs appears to be associated with a variety of cancers (57, 58). For example, miR-21 was first discovered as an oncomiRNA due to its universal overexpression in a variety of cancers (57, 58). Aberrant biosynthetic process of miRNAs (e.g., miR-21) has also been shown to be involved in the production of oncomiRNAs (58, 59). In addition, miRNA genes are frequently subjected to epigenetic changes in cancer leading to tumor progression (56). Furthermore, using a systematic miRNA inhibitor treatment technique on cancer cells, Ma et al discovered miR-10b overexpression which is required for tumor migration and invasion in metastatic breast cancer cells (60). Interestingly, unique miRNA such as miR-302 appears to play a key role in the maintenance of stemness properties in normal stem cells and in cancer stem cells (61). Aside from the abnormal biosynthetic processes and epigenetic modifications of miRNA genes, it has become apparent recently that tight interactions between certain miRNAs and transcription factor-mediated regulatory circuits may also influence important biological outcomes and drives cellular transformation (57, 58). Nevertheless, the abnormal signaling pathways responsible for the onset of oncogenic miRNAs during cancer development and progression remains poorly understood. In this review article, I plan to focus on several HA-CD44 interaction-induced oncogenic signaling pathways that regulate several miRNAs and downstream effector functions in a variety of cancer cells during tumor progression.

### Regulation of miR-21 Signaling by HA-CD44 Interaction in Cancers

Upregulation of miR-21 has been detected in tumors and to a lesser extent in normal tissues (58, 59). In recent years, miR-21 has received a great of attention due to the discovery of its specific targets and functional involvement in cancer progression (62, 63). For example, the gene expression of program cell death (PDCD4, a tumor suppressor protein) can be blocked by miR-21 (62, 63). This miR-21-medited downregulation of PDCD4 results in tumor progression (62–64). Therefore, miR-21 can be viewed as a cancer cell activator. During HA/CD44 signaling, miR-21 has also been suggested to regulate tumor cell proliferation, invasion, survival, chemoresistance and tumor progression (19, 62–67). The oncogenic signaling pathways involved in the regulation of miR-21 and its function by HA-CD44 interaction in solid tumor cancers are described below:

#### The Expression of miR-21 and Nanog-DROSHA-p68 Signaling

Several reports showed that the interaction between RNase III DROSHA/RNA helicase p68 (DROSHA/p68) and other regulatory molecules plays an important role in regulating the biogenesis of miRNAs (68). It has been shown that p53 and the DROSHA form complexes with the RNA helicases (p68/p72) during miRNA production in HCT116 cells (68). It is also documented that TGFβ-mediated SMAD-2 signaling promotes miR-21 expression (69). Specifically, DROSHA-p68 complex promotes the biogenesis of miR-21 by converting pri-miR-21 into pre-miR-21 during TGFβ-specific SMAD signaling events (69). Thus, it is apparent that the production of miR-21 is closely regulated by the DROSHA/p68 microprocessor complex during cellular signaling.

**Figure 3:**
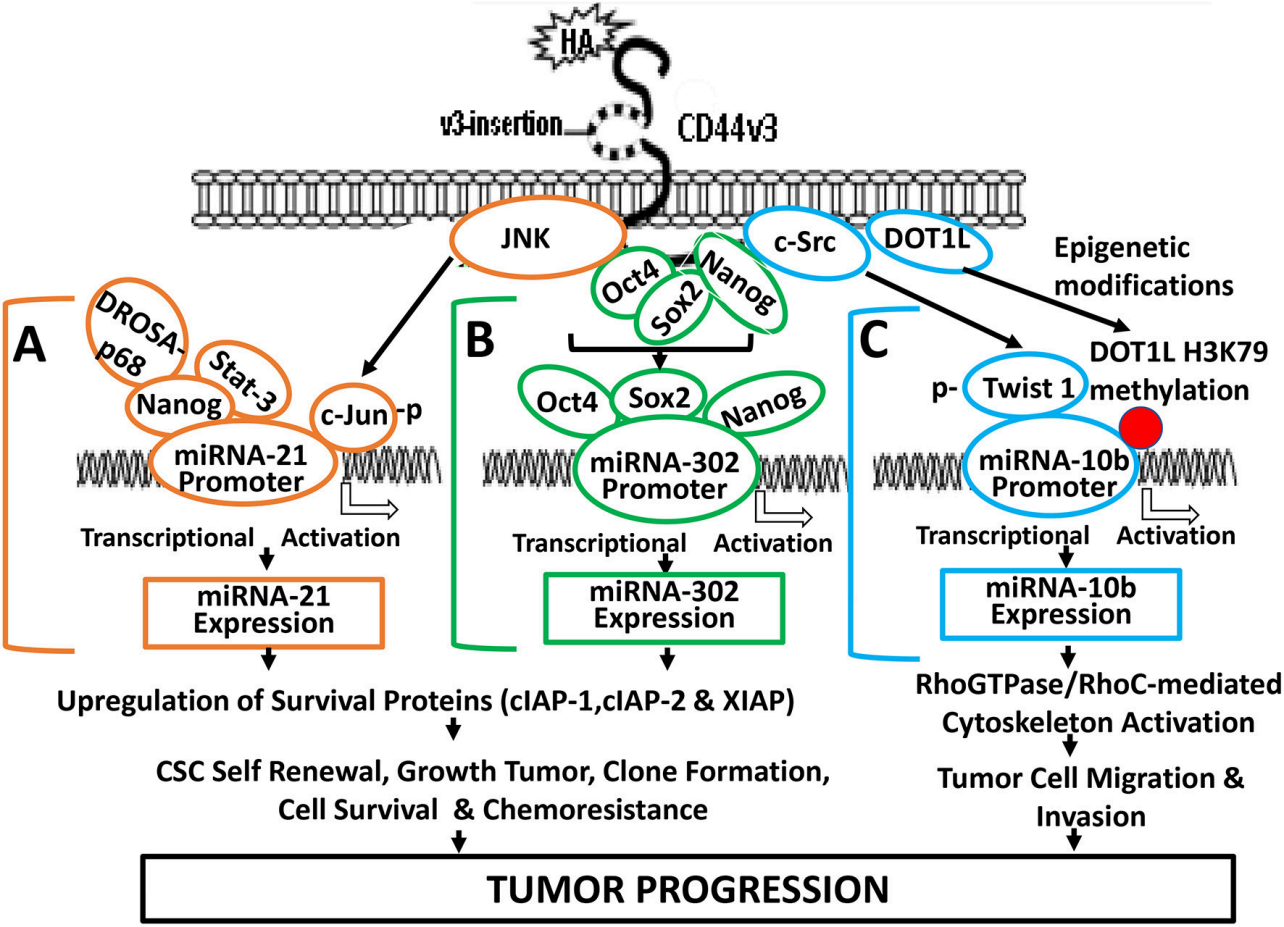
A proposed model for HA-CD44-mediated signaling activation in the regulation of miRNA-21, miR-302, and miR-10b production and oncogenesis in tumor cells. **(A)** The binding of HA to CD44 promotes JNK activity which, in turn, causes phosphorylation of c-Jun. Phosphorylated c-Jun then binds to the miR-21 promoter and induces miR-21 expression. HA binding to CD44 also causes Nanog interaction with Stat-3 and the microprocessor complex containing the RNase III (DROSHA) and the RNA helicase (p68). These Nanog-associated signaling complexes (containing Stat-3 and/or DROSHA and p68) then bind to miR-21 promoter region, resulting in miR-21 production leading to upregulation of IAP protein expression, tumor cell survival, and chemoresistance. **(B)** HA-CD44 interaction promotes miRNA-302 expression and chemoresistance: HA binding to CD44 promotes an association between CD44v3 and OCT4/SOX2/Nanog. Subsequently, OCT4/SOX2/Nanog complexes interact with the promoter region (containing OCT4-, SOX2-, and Nanog-binding sites) of the miR-302 cluster resulting in miR-302 cluster gene expression and mature miR-302 production. The resultant miR-302 then functions to induce IAP (cIAP-1, cIAP-2, and XIAP) expression, tumor cell growth, self-renewal, clone formation, tumor cell survival, and chemoresistance in tumor cells. **(C)** HA-CD44 interaction promotes miRNA-10b expression and tumor migration/invasion: HA binding to CD44 promotes c-Src phosphorylation (kinase activation), which, in turn, causes phosphorylation of Twist. Phosphorylated Twist then interacts with the E-box elements of mR-10b promoter, resulting in miR-10b gene expression, and mature miR-10b production. The binding of HA to CD44 also enhances DOT1L upregulation and DOT1L/H3K79 methylation-mediated epigenetic changes, resulting in methyl-H3K79 binding to miR-10b promoter, and miR-10b gene expression/production. The expressed miR-10b then promotes upregulation of RhoGTPase-mediated cytoskeleton activation leading to tumor cell migration and invasion. Red dot represents DOT1L/H3K79-mediated histone modifications (via epigenetic changes).

HA/CD44 activated stem cell marker (Nanog) signaling pathways are also involved in regulating miR-21 expression in both breast and head and neck cancer cell lines (19, 65–67). For example, HA binding to CD44 promotes Nanog association with DROSHA/p68 microprocessor complex resulting in the upregulation of miR-21 and downregulation of PDCD4 (a tumor suppressor protein) in cancer cells. Consequently, several inhibitors of apoptosis proteins (IAPs) (e.g., c-IAP-1, cIAP-2, and XIAP) are also upregulated resulting in anti-apoptosis and chemotherapy resistance (Figure 3A). The knowledge obtained from this biogenesis study of miR-21 regulated by Nanog-DROSHA-p68 complexes may provide useful foundation for designing new drug target to downregulate miR-21 and increase tumor cell death and enhance chemosensitivity for the treatment of HA/CD44-mediated cancer.

#### The Expression of miR-21 and Nanog-Stat-3 Signaling

Abnormal Stat-3 signaling are well-known to play important roles in oncogenesis (70). Constitutively activated Stat-3 has been closely associated with human malignancies (46). It has been shown that Nanog and Stat-3 are functionally coupled in many cancer cells (39) (Figure 3A). For example, HA binding to CD44 induces a physical association between Nanog and Stat-3 in head and neck cancer cells leading to miRNA-21 gene expression and production (66). Most importantly, treatments of cancer cells with several signaling perturbation agents such as Nanog siRNA or Stat-3siRNA or an anti-miR-21 inhibitor result in downregulation of survival proteins (e.g., cIAP-1, cIAP-2, and XIAP) and upregulation of PDCD4 leading to tumor cell apoptosis/death and chemosensitivity in head and neck cancer (19, 65–67). Thus, this newly-discovered Nanog-Stat-3-regulated miR-21 signaling pathways during HA-CD44 interaction may be considered as another new drug target to treat cancers.

#### The Expression of miR-21 and JNK/c-Jun Signaling

Induction of oncogenic signaling frequently involves abnormal JNK-regulated c-Jun activities (71, 72). The transcription factor, c-Jun belongs to the AP-1 family which has been shown to play an important role in regulating cell transformation (73). Specifically, c-Jun has been shown to regulate the expression of p53 and cyclin D1 (73, 74) and has also been shown to accelerate leukemogenesis by activating cell cycle-related genes in cancer cells (73). JNK-regulated c-Jun often functions as a “bodyguard” which prevents certain gene modification(s) during cancer-related process (73–76). A previous study showed that c-Jun involves transcriptional activation of miR-21 at the miR-21 promoter region located at AP-binding sites (76). HA-CD44 binding has also been shown to cause miR-21 production in a JNK/c-Jun-dependent manner in breast tumor cells (67) (Figure 3A). We have found that inhibition of JNK/c-Jun-induced miR-21 signaling by various signaling perturbation agents such as JNK inhibitor or c-Jun siRNA or anti-miR-21 inhibitor effectively downregulates the expression of survival proteins such as Bcl2 and IAP family of proteins leading to apoptosis/cell death and chemosensitivity. These findings strongly suggest that JNK/c-Jun-regulated miR-21 activated by HA-CD44 interaction plays a pivotal role in tumorigenesis and drug resistance. Consequently, it is possible to design therapeutic drugs to target JNK/c-Jun-regulated miR-21 for the treatment of HA/CD44-mediated cancer.

### HA-CD44-Mediated miR-10b Signaling in the Regulation of Tumor Cell Migration and Invasion

Tumor-specific phenotypes (e.g., tumor cell migration, invasion and metastasis) are often regulated by oncogenic signaling processed and/or cytoskeleton functions (1). Overexpression of miRNA-10b has been shown to be closely associated with upregulation of RhoC during glioma invasion and migration (77). In addition, the expression of a zinc finger protein, KLF4 (Kruppel-like factor 4) was found to be regulated by miR-10b in certain cancer cell lines (78). Moreover, it has been reported that miR-10b is responsible for activating both tumor cell invasion and metastasis (79). HA appears to interact with CD44 and induces miR-10b expression in head and neck cancer cells (32, 33). Interestingly, 200 kDa-HA fragments (to a lesser extent 5 kDa, 20 kDa, or 700 kDa) appears to preferentially enhances miR-10b expression in CSCs from head and neck cancer cells (32). The level of miR-10b expression is significantly higher (at least 5–10-fold increase) than other miRNAs (e.g., miR-373, 27b,181-miRNA, miR-34b, and miR-145) detected in 200 kDa-HA-treated head and neck cancer cells (32). Here, several HA/CD44-mediated miR-10b signaling events and functions in various cancers will be described below:

#### c-Src and Twist Signaling in the Regulation of miR-10b Expression

Src kinase family members (e.g., Lck, Yes, and Fyn) have been shown to participate in CD44-mediated cellular signaling processes (80–82). For example, during T-cell activation Lck is found to be tightly linked to CD44 (80). Both Lck and Fyn have also been shown to be closely complexed with CD44 in a specialized plasma membrane domain enriched in glycosphingolipid in lymphoid cells (81). Moreover, CD44 has been shown to form a tight association with other Src kinase family of proteins (e.g., c-Src, Yes, and Fyn) during abnormal prostate cancer cell proliferation and growing processes (82). These findings strongly support the notion that CD44 and certain c-Src kinases family members are physically linked and functionally coupled.

Twist (one of c-Src substrates) has been shown to promote a variety of tumor cell-specific functions (e.g., EMT transition, invasion and drug resistance) (83–85). Twist is also considered as a putative oncogene for its role in regulating CD44-expressing breast cancer stem cells (CSCs) (86). Several Twist-regulated oncogenic events have been reported to be regulated by the binding of Twist to the promoters (containing the E-boxes) of specific genes (e.g., E-cadherin) required for tumor cell survival and invasiveness (87) as well as transcriptionally repression of E-cadherin gene expression in breast cancer (88). Previous studies showed that c-Src-activated Twist promotes miR-10b expression in breast tumor cells (79, 89). During HA/CD44-mediated signaling process, Twist phosphorylated by c-Src is also able to interact with miR-10 promoter (with E-box domain) and activates the onset of miR-10b gene expression/production and tumor cell-specific activities in cancer cells (89). Treatment of cancer cells with c-Src inhibitor, PP2, or Twist siRNA significantly blocks the production of HA/CD44-mediated miR-10 expression and downstream RhoGTPase (RhoC)-ROK effector functions (89). These observations strongly suggest that HA-CD44 interaction promotes miR-10b expression required for tumor cell-specific functions (e.g., cytoskeleton-associated metastasis, invasion, and metastasis) in a c-Src/Twist-dependent manner.

#### Role of Epigenetic Modifications in Regulating miR-10b Expression

Epigenetic regulation via histone methylation participates in modifying chromatin organization together with reprogramming gene expression during cancer progression (90). The histone methyltransferase, DOT1 is known to be solely responsible for catalyzing methylation of histone at lysine 79 residues in three different ways such as H3K79me1/H3K79me2/ H3K79me3 in budding yeast, *Saccharomyces cerevisiae* (91, 92). Mammalian DOT1 (so-called DOT1L) has also been documented to display an ability to conduct histone methylation at lysine 79 residues as methyltransferases involved in modifications of gene expression (93). Both histone methyltransferases play an important role in H3K79 methylation involved in transcriptional regulation for the DNA damage checkpoint, meiotic checkpoint and cell cycle progression (94). Abnormal DOT1L-mediated H3K79 methylation has also been detected in mixed lineage leukemia (MLL) (95). In addition, suppression of DOT1L expression causes a reduction of tumor cell growth (96). These findings all indicate that histone methyltransferase (e.g., DOT1L) is closely involved in the development of cancer. DOT1L-mediated methylation of histone H3 at lysine 79 (H3K79) is also involved in the development of embryonic stem (ES) cells (97). Recent studies indicate that the activity of histone methyltransferase (e.g., DOT1L) can be detected in HA-activated head and neck cancer stem cells (CSCs) (89). Specifically, HA promotes DOT1L-regulated H3K79 methylation of miR-10b promoter binding sites leading to miR-10 production resulting in CSC-specific functions in head and neck cancer (Figure 3C). Silencing of DOT1L with DOT1LsiRNA and/or miR-10b with antagomirs (an anti-miR-10 inhibitor) significantly decreases the amount of miR-10b production resulting in downregulation of RhoC expression and tumor cell migration/invasion (66, 74). These findings may provide ground work for the development of new therapeutic drugs to target either DOT1L or miR-10 for the treatment of HA/CD44-activated cancer.

### Nanog/Oct4/Sox2 Signaling in Regulating miR-302 Expression and Cancer Stem Cell (CSC) Activation and Chemotherapy Resistance

The miR-302 family which encodes a cluster of eight miRNAs has been shown to be important in the “stemness” properties of either normal and abnormal stem cells (98–100). These observations strongly suggest that there is a close involvement of miR-302 in the regulation of pluripotency of stem cells. The transcription factors such as Nanog, Oct4, and Sox2 often interact with each other during transcriptional events (61, 100, 101). Oct4, Sox2, and Nanog have also been detected to co-occupy the promoter sites of miR-302 for the activation of target genes required for development and oncogenesis (61, 100, 101). In addition, miR-302 family plays key roles in regulating cell proliferation and cell fate determination during differentiation at the post-translational level (61, 100, 101). A previous study showed that HA binding to CD44 promotes the expression of miR-302 in a Nanog/Oct4/Sox2-dependent manner in head and neck cancer stem cells (CSCs) (31) (Figure 3B).

Several miR-302 downstream targets such as AOF1 and AOF2 known as lysine-specific histone demethylases have been shown to play a role in demethylating H3K4 and inhibiting transcription of genes (31, 102, 103). Suppression of AOF1 and AOF2 is known to induce DNA (cytosine-5)-methyltransferase 1 (e.g., DNMT1) degradation and global demethylation leading to reprogramming of somatic cells into induced pluripotent stem cells (31). HA-CD44-activated miR-302 has also been shown to cause DNMT1 reduction and DNA demethylation in CD44v3-expressing cancer stem cells (CSCs) (31). Moreover, this DNA demethylation process regulated by HA-CD44-activated miR-302 can activate the expression of several Inhibitor of Apoptosis Protein (IAP) family of proteins such as c-IAP1, c-IAP2, and XIAP which appear to be closely linked to several important activities unique for cancer stem cells (CSCs) isolated from head and neck caner (31) (Figure 3B). Most importantly, treatments of CSCs with anti-miR-302 inhibitors readily upregulate lysine-specific histone demethylases and reduces DNA global demethylation as well as impairs HA/CD44-activated CSC functions (79). It is likely that miR-302 signaling pathway regulated by stem cell markers such as Nanog/Oct4/Sox2 during HA-CD44 interaction may be used as a novel therapeutic drug target to downregulate cancer stem cell (CSC) functions and to overcome chemotherapy resistance in cancer cells.

## HA-CD44 Interaction in Stimulating lncRNA (UCA1) Signaling and Tumor Progression

The evolutionarily conserved long non-coding RNAs (so-called lncRNAs >200 nucleotides) are now recognized as a major component of the human transcriptome (104, 105). Most of these molecules remain to be functionally unknown (104, 105). Dysregulation of lncRNAs frequently involves alterations of transcriptional and post-transcriptional activities of gene regulation in many cancers (106–111). For example, downregulation of PTCSC3 was detected in thyroid cancers (112). Malfunction of HULC and XIST is also reported in various cancers (113–118). Furthermore, both GAPLINC and MALAT1 have been used as unfavorable predictors for a few solid tumor cancers (119–123). Overexpression of HOTAIR is linked to metastasis in colorectal, liver, pancreatic, breast and gastric cancers (124–131) whereas ANRIL and PRNCR1 upregulation is detected in prostate cancer (132, 133). High levels of KCNQ1OT1 and H19 expression were also detected in colorectal cancer (132) and hepatocellular carcinoma (133), respectively. Therefore, aberrant expression of certain lncRNAs appears to be closely linked to various tumor progression.

Another important member of lncRNA family, urothelial carcinoma associated 1 (lncRNA UCA1) has been shown to be correlated with tumor growth, progression and recurrence (108–111). Several studies focusing on the transcriptional regulation of lncRNA UCA1 show that many transcription factors (e.g., C/EBPα, Ets-2, TAZ/YAP/TEAD, HIF-1α, SATB1, CAPERα/TBX3, etc.) may participate in the regulation of lncRNA UCA1 by binding to the promoter sites of lncRNA UCA1 (134–139). A specific example for the regulation of lncRNA UCA1 expression by certain transcription factor during HA-CD44 interaction in head and neck cancer cells is described as follows:

### Role of C/EBPα in Regulating HA-CD44-mediated lncRNA UCA1 Expression

**Figure 4:**
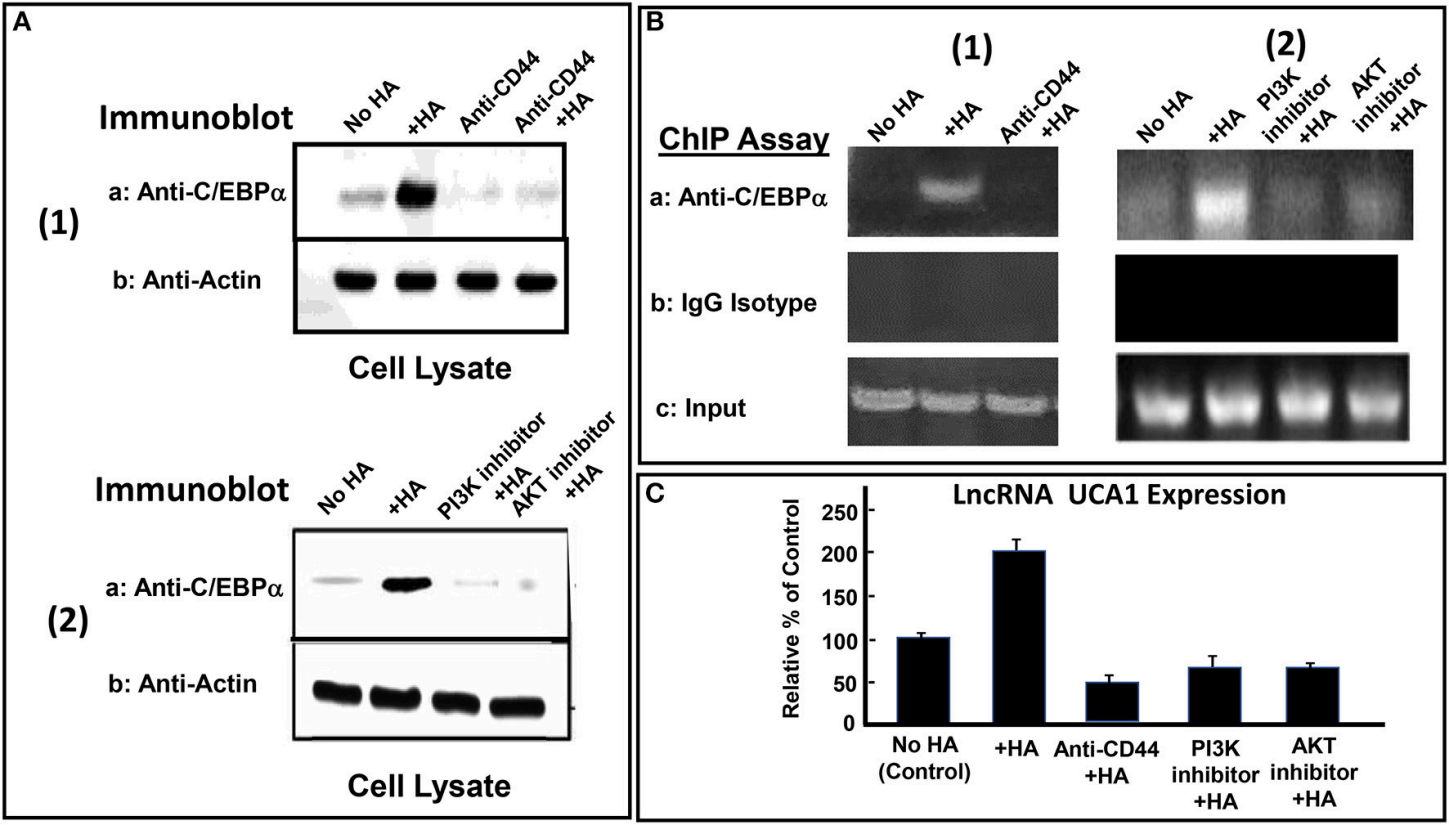
HA-CD44-mediated LncRNA UCA1 expression in tumor cells. **(A-1)** Detection of C/EBPα phosphorylation in CD44v3high HSC-3 cells treated with no HA, (lane 1); or with HA (lane 2); or with anti-CD44 antibody with no HA (lane 3); or with anti-CD44 antibody + HA (lane 4). **(A-2)** Detection of C/EBPα phosphorylation in CD44v3high HSC-3 cells treated with no HA, (lane 1); or with HA (lane 2); or with PI3K inhibitor (GDC-0941) plus HA, (lane 3); or with AKT inhibitor (GSK795) plus HA (lane 4). **(B)** ChIP assay of p-C/EBPα binding to lncRNA UCA1 promoter in CD44v3high HSC-3 cells treated with no HA (lane 1) or with HA (lane 2) or with PI3K inhibitor (GDC-0941) plus HA (lane 3) or with AKT inhibitor (GSK795) plus HA (lane 4) using anti-p-C/EBPα or IgG control. Co-immunoprecipitated DNA was amplified by PCR with primers specific for the lncRNA *UCA1* promoter. **(C)** The expression of lncRNA *UCA1* by qRT-PCR in CD44v3high head and neck cancer cells (HSC-3 cells) treated with no HA (bar 1) or with HA (bar 2) or with anti-CD44 antibody plus HA (lane 3) or with PI3K inhibitor (GDC-0941) plus HA (bar 4) or with AKT inhibitor (GSK795) plus HA (bar 5) using lncRNA *UCA1*-specific primers and Q-PCR assay.

Many transcription factors have been shown to be involved in the regulation of lncRNA UCA1 expression (135). For example, the interaction between the transcription factor, C/EBPα and the promoter of lncRNA UCA1 often promotes an upregulation of lncRNA UCA1 production leading to anti-cell death and cell survival (135). Recent study indicates that a cross-talk between PI3K-AKT pathway and lncRNA UCA1 expression also occurs during breast cancer cell invasion (140). We have found that HA induces C/EBPα phosphorylation in CD44v3high head and neck cancer cells (HSC-3 cells) in a CD44-dependent manner (Figure 4A-1). Downregulation of AKT by treating CD44v3high HSC-3 cells with AKT inhibitor (GSK795) or PI3K inhibitor (GDC-0941) blocks HA-mediated C/EBPα phosphorylation (Figure 4A-2). These findings suggest that C/EBPα phosphorylation is PI3K-AKT signaling-dependent in HA-treated CD44v3high head and neck cancer cells (HSC-3 cells) (Figures 4A-1, 2). To examine whether phosphorylated C/EBPα (induced by HA-mediated CD44v3-mediated PI3K-AKT activation) directly interacts with the promoter region of lncRNA UCA1, chromatin immunoprecipitation (ChIP) assays were performed in head and neck cancer cells with HA (or without HA). Preliminary data indicate that phosphorylated C/EBPa is directly recruited into the promoter region of lncRNA UCA1 in HA-treated CD44v3high head and neck cancer cells, resulting in lncRNA UCA1 expression (Figure 4B–1). However, HA-mediated recruitment of phosphorylated C/EBPa into LncRNA UCA1 promoter sites appears to be blocked in cells treated with anti-CD44 antibody (Figure 4B-1). Consequently, lncRNA UCA1 expression is also inhibited (Figure 4C). Downregulation of PI3K or AKT by treating cells with either PI3K inhibitor (GDC-0941) or AKT inhibitor (GSK795) effectively inhibits the complex formation between phospho-C/EBPa and the promoter region of lncRNA UCA1 in HA-treated CD44v3high head and neck cancer cells, as well as lncRNA UCA1 production (Figure 4B-2 and Figure 4C). These findings suggest that the binding of phosphorylated C/EBPa to the lncRNA UCA1 promoter and lncRNA UCA1 expression is CD44/PI3/AKT-dependent and GDC-0941/GSK795-sensitive in HA-treated CD44v3high head and neck cancer cells. Therefore, we believe that the regulation of lncRNA UCA1 expression in head and neck cancer cells is HA-dependent and CD44-specific.

### Role of lncRNA UCA1 in Regulating Tumor Cell Survival and Chemoresistance

Several regulatory small and long non-coding RNAs have been well-documented in many cancers resistant to therapeutic drug (e.g., cisplatin) treatment (141–144). The ability of cisplatin to induce tumor cell death is often counteracted by the presence of anti-apoptotic regulators and/or survival proteins leading to chemoresistance (142–144). The IAP family (e.g., cIAP-2 and XIAP) is well-documented to play critical roles in promoting tumorigenesis through the action of both anti-apoptosis and anti-cell death (145). These proteins also participate in chemoresistance by reducing tumor cell death or apoptosis caused by chemotherapeutic drugs (146). Although several other survival proteins such as Bcl2 and BclxL are known to play roles in regulating tumor cell survival and chemoresistance in cancer cells during HA-CD44 binding (147), the involvement of cIAP-1, cIAP-2, and XIAP in promoting HA/CD44-mediated tumor cell survival and drug resistance has only recently received some attentions.

**Figure 5:**
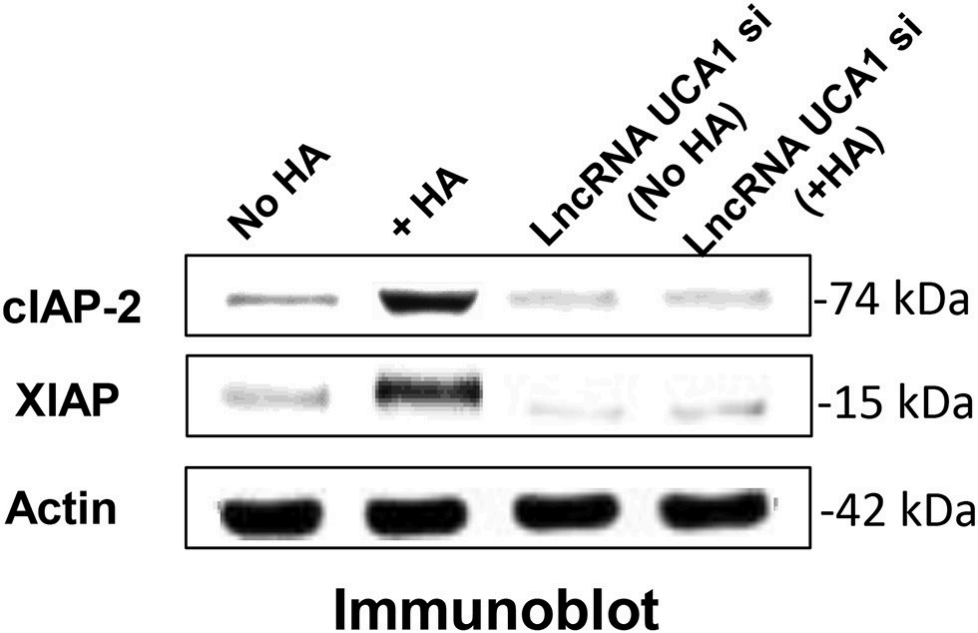
Detection of LncRNA UCA1 effect on HA/CD44-induced survival protein expression in cancer cells (HSC-3 cells) with no HA or with HA or treated with lncRNA UCA1 si with no HA or with HA.

LncRNA UCA1 has been reported to induce drug resistance in bladder cancer and many other cancers (108, 109, 148–163), thereby greatly reducing the efficacy of cancer therapy. Recently, we found that the expression of both cIAP-2 and XIAP appear to be downregulated in head and neck cancer cells treated with anti-lncRNA UCA1 inhibitor (Figure 5). The suppression of these survival proteins leads to tumor cell death and effective chemotherapeutic drug treatment (Table 1). The fact that reduction of anti-apoptosis proteins (cIAP-2 or XIAP) by treating head and neck cancer cells with specific inhibitory siRNAs (e.g., cIAP-2 siRNA or XIAP siRNA) during HA/CD44 interaction appears to increase chemosensitivity suggests that downregulation of lncRNA UCA1 together with blockage of survival protein pathways may provide a new therapeutic strategy in cancer therapy, especially dealing with drug resistance.

**Table 1:** Chemosensitivity assay treatment.

**Chemosensitivity assay treatment**	Growth inhibition IC50 (μM)^*^
No HA (Control)	1.00 ± 0.05
+HA	2.69 ± 0.11
LncRNA UCA1si-treated cells (+HA)	0.50 ± 0.07
cIAP-2 siRNA-treated cells (+HA)	0.42 ± 0.04
XIAP siRNA-treated cells (+HA)	0.45 ± 0.02

* *The procedures for measuring cisplatin-induced tumor cell growth inhibition (IC 50) is the same as described previously (31–33)*.

### The Role of lncRNA UCA1 in Regulating miR-145-ROCK1 Pathway and Tumor Cell Migration and Invasion

During the regulation of miRNA expression, lncRNA can compete the common response elements of miRNAs (161). LncRNAs can also bind DNAs, RNAs and proteins by acting as decoy, guide or scaffold (161). LncRNA has been reported to bind to many different miRNAs including miR-145 in a variety of cancer cells resulting in carcinogenesis, tumor cell migration, invasion or drug resistance (152, 153, 162–167, 167–169). Recent observations indicate that lncRNA UCA1 promotes migration and invasion in bladder cancer cells (164). Accumulating evidence indicates that miR-145 known as a tumor suppressor is frequently downregulated in various cancers (170). It has been postulated that a signaling pathway may be involved in the formation of a regulatory loop between lncRNA UCA1 and miR-145 via a reciprocal repression process required for tumor cell-specific activities (e.g., migration and/or invasion) (164). There is evidence that upregulation of miR-145 impairs cancer cell motility by downregulating the expression of its target genes including ROCK (a Rho-associated protein kinase), a key regulator of actin cytoskeleton reorganization required for cancer cell migration and invasion (164, 171). Thus, both up- or down-regulation of miR-145 appear to play important roles in cancer cell-related activities (e.g., cell migration and invasion).

**Figure 6:**
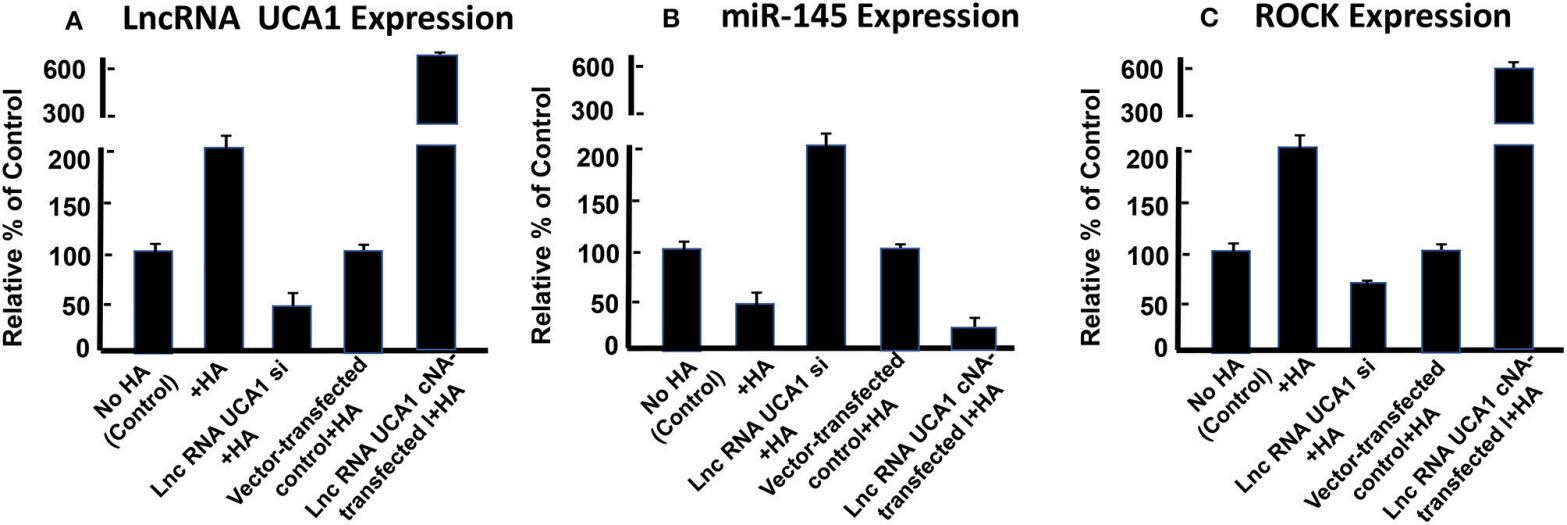
Detection of HA/CD44-mediated expression of lncRNA UCA1 **(A)**, miR-145 **(B)**, and ROCK **(C)** by qRT-PCR in CD44v3high head and neck cells (HSC-3 cells) treated with no HA (bar 1) or with HA (bar 2) or transfected with si lncRNA UCA1 plus HA (bar 3) or transfected with pcDNA 3.1 plus HA (bar 4) or transfected with pcDNA 3.1-lncRNA UCA1 plus HA (bar 5).

Furthermore, it has been reported that lncRNA UCA1 suppresses the tumor suppressor miR-145 for tumor cell invasion/migration through the expression of miR-145 target proteins such as ROCK1 in glioma cancer cells (109). These findings are consistent with our observations showing that HA-CD44v3 interaction stimulates lncRNA UCA1 expression in CD44v3high tumor cells (Figure 6A). Moreover, upregulation of lncRNA UCA1 (by transfecting cells with lncRNA UCA1 cDNA, but not vector control cDNA) significantly suppresses miR-145 expression (Figure 6B) leading to an increase of miR-145 target gene, ROCK1 expression (Figure 6C). Conversely, when CD44v3high head and neck cancer cells (HSC-3 cells) were transfected with lncRNA UCA RNAi inhibitor, the expression level of miR-145 is significantly up-regulated (Figure 6B). Consequently, ROCK1 (a miR-145 target) gene/protein is downregulated (Figure 6C). These findings suggest that the miR-145-ROCK1 pathway serves as a possible downstream functional target for lncRNA UCA1 in CD44v3high head and neck cancer cells. Furthermore, our recent data show that upregulation of lncRNA UCA1 by HA-CD44v3 binding in CD44v3high head and neck cancer cells significantly enhances ROCK-mediated head and neck cancer cell migration and invasion (Table 2). Treatment of cells with lncRNA UCA1 RNAi inhibitor or miR-145 mimic or a ROCK inhibitor, Y-27632 significantly reduces tumor cell invasion (Table 2). These observations suggest that the miR-145-ROCK1 pathway serves as a new downstream functional target for lncRNA UCA1 in regulating CD44v3high head and neck cancer cell activities including cell migration and invasion. The results of these studies strongly indicate that lncRNA LncRNA UCA1 is one of the important regulatory molecules in controlling miR-145-ROCK pathway and tumor cell migration and invasion.

**Table 2:** Cell migration and invasion treatments.

**Cell migration and invasion treatments**	**Tumor cell migration (% of control)**	**Tumor cell invasion (% of control)**
No HA (Control)	100 ± 2	100 ± 5
+HA	223 ± 12	245 ± 10
LncRNA UCA1 si-treated (+HA)	65 ± 3	63 ± 2
miR-145 mimic-treated cells (+HA)	65 ± 2	62 ± 2
ROCK inhibitor (Y-27632)-treated cells (+HA)	60 ± 2	66 ± 3

*The measurements of in vitro tumor cell migration and invasion were performed using twenty-four transwell units as described previously (66, 131, 154)*.

**Figure 7:**
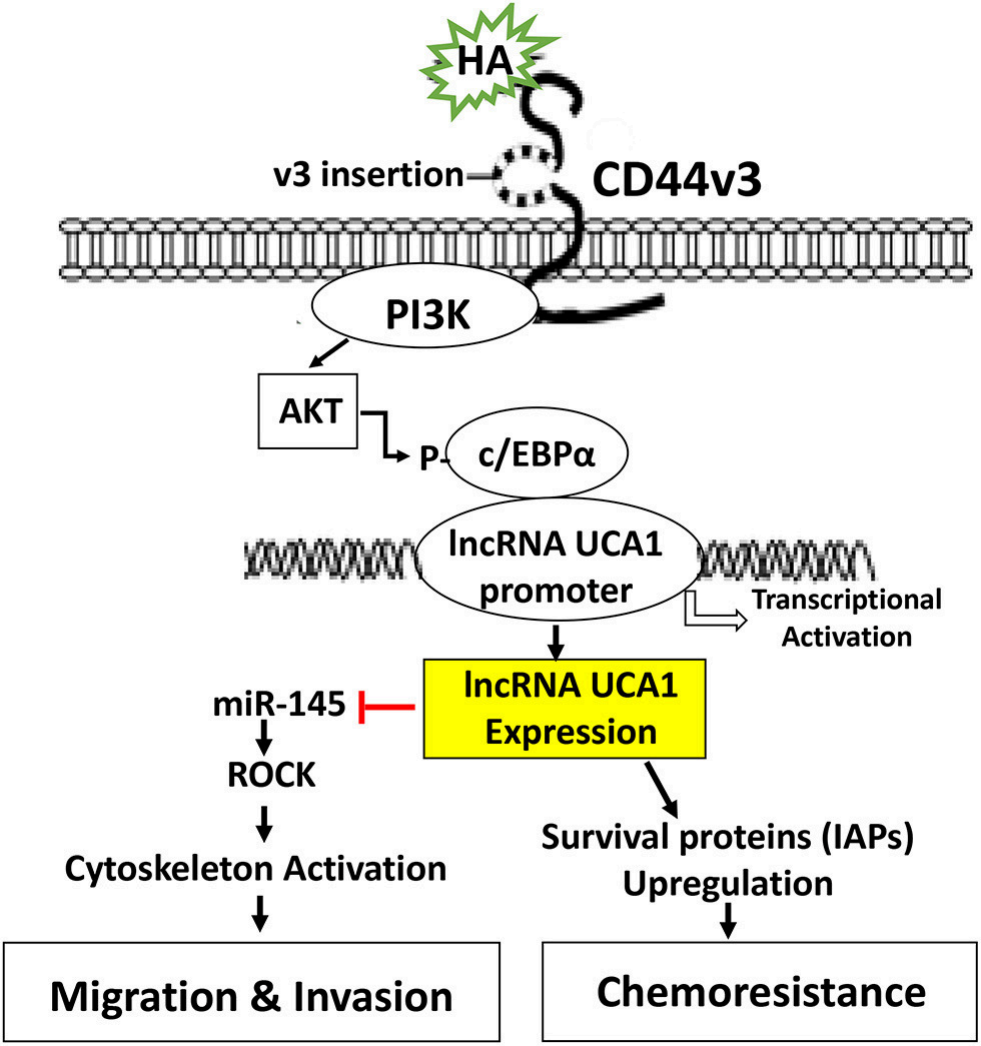
A proposed model for HA-CD44-mediated signaling activation in the regulation of LncRNA UCA1 production and oncogenesis in tumor cells. The binding of HA to CD44 stimulates PI3K and AKT signaling which in turn causes c/EBPα phosphorylation. Phosphorylated c/EBPα then binds to the promoter of LncRNA UCA1 and induces transcriptional activation for LncRNA UCA1 expression. The production of LncRNA UCA1 upregulates survival proteins (IAPs) and chemoresistance. LncRNA UCA1 also downregulatemiR-145 expression and stimulates ROCK expression and cytoskeletal activation leading to tumor cell migration and invasion.

In summary, we would like to propose that HA binding CD44 stimulates PI3K and AKT signaling which in turn causes c/EBPα phosphorylation. Phosphorylated c/EBPα then binds to the site of LncRNA UCA1 promoter and induces transcription activity for the expression of LncRNA UCA1 which then upregulates survival proteins (IAPs) and chemoresistance. HA/CD44-activated lncRNA UCA1 also downregulates miR-145 expression and stimulates ROCK expression required for cytoskeletal activation and tumor cell motility (e.g., migration and invasion) (Figure 7). Therefore, it is feasible to use either LncRNA UCA1 si treatment and/or ROCK inhibitor to limit tumor cell migration and invasion (Table 2) and to reduce HA/CD44-induced tumor metastasis and progression.

## Conclusion

**Figure 8:**
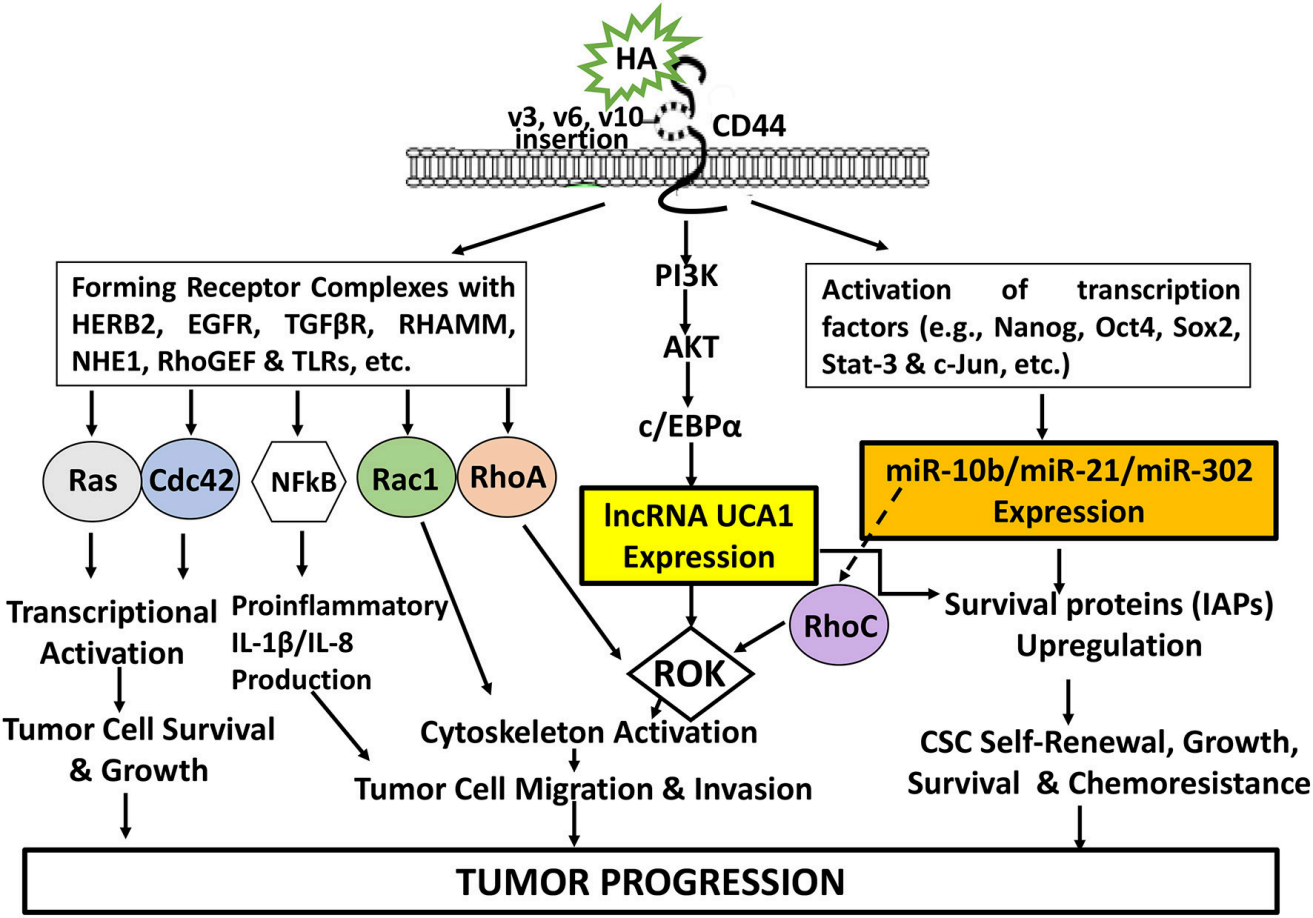
An Illustration of HA-CD44 interaction-induced oncogenic signaling events in cancer. The binding of HA (large vs. small HA) to CD44v isoforms interaction stimulates CD44-other receptor (HERB2, EGFR, TGFβR, RHAMM, NHE1, RhoGEF, and TLRs, etc.) complex formation and Ras/Cdc42/Rac1/RhoA/NFkB activation for transcriptional activation and proinflammatory cytokine/chemokine production as well as tumor cell survival, growth, invasion and migration. HA-CD44 interaction also promotes PI3K/AKT activation and LncRNA (UCA1) production resulting in ROK-mediated cytoskeleton function required for tumor cell migration and invasion. Moreover, HA/CD44 activates transcriptional factor-induced miRNA (miR-10b/miRNA-21/miR-302) expression leading to CSC self-renewal, growth, survival and chemoresistance. Furthermore, the induction of miR-21 by HA-CD44 interaction also stimulates RhoC upregulation and ROK-regulated tumor cell migration and invasion. All these events contribute to HA-CD44 interaction-mediated tumor progression.

The newly discovered signaling events regulated by HA-CD44 interaction may be very useful for a better understanding of cancer cell-specific behaviors including transcriptional activation, tumor cell growth, inflammatory cytokine/chemokine production, migration/invasion and survival as well as chemoresistance as summarized in Figure 8. Consequently, targeting CD44 using anti-CD44 and/or CD44 variant-specific antibody and/or anti-sense strategies to downregulate CD44 and/or CD44 variants may be a possible choice for the development of new cancer cell-based therapies. Furthermore, HA-based nanoparticles containing therapeutic drugs (e.g., cisplatin or doxorubicin) may be used to accurately deliver therapeutic drugs into CD44v isoform-expressing cancer cells to enhance chemo-sensitivity and downregulate CD44v isoform-mediated oncogenic signaling. It is also feasible to design signaling perturbation strategies to downregulate the expression of HA/CD44-regulated Nanog/Oct4/Sox2 and c-Jun as well as certain miRNAs (e.g., miR-21, miR-10b, and miR-302) using specific inhibitors such as siRNA and shRNA and anti-miRNA inhibitor approaches to simultaneously suppress both oncogenic behaviors and cancer progression. Since many LncRNAs have been shown to be closely associated with tumor cell-specific properties including cell survival, chemoresistance, tumor cell migration and invasion, it will also be possible to develop novel signaling perturbation techniques to simultaneously inhibit both oncogenic miRNAs and LncRNA UCA1 using miRNA-21/miRNA-10b/miR-302 RNAi inhibitor and/or lncRNA UCA1 RNAi inhibitor treatments. These strategies could synergistically cause apoptotic responses and chemosensitivity. These new approaches could indicate that the impairment of specific signaling pathways together with suppression of miRNAs (miR-21/miR-10b/miR-302) and/or lncRNA UCA1 in HA-CD44-activated cancer cells may be more effective than chemotherapy alone. Novel therapeutic strategies described in this review may offer helpful information for understanding the initiation and development mechanisms of different cancers comprehensively and suggest new therapeutic targets for clinical treatment of HA/CD44-activated cancer development and tumor progression.

## Author Contributions

The author confirms being the sole contributor of this work and has approved it for publication.

### Conflict of Interest Statement

The author declares that the research was conducted in the absence of any commercial or financial relationships that could be construed as a potential conflict of interest.
